# Data for the elaboration of the CIPROS checklist with items for a patient registry software system: Examples and explanations

**DOI:** 10.1016/j.dib.2017.07.075

**Published:** 2017-08-03

**Authors:** Doris Lindoerfer, Ulrich Mansmann

**Affiliations:** Institut für medizinische Informationsverarbeitung, Biometrie und Epidemiologie (IBE), Ludwig-Maximilians-Universität München, Marchioninistraße 15, D-81377 München, Germany

**Keywords:** Checklist, Patient registry software systems, Examples and explanations

## Abstract

The data presented relates to the publication “Enhancing Requirements Engineering for Patient Registry Software Systems with Evidence-based Components” (Lindoerfer and Mansmann, 2017) [1], which describes the strategy behind the development of the CIPROS checklist. This manuscript also compares CIPROS with general requirements specification templates, and standards. The data is shortly described in Section 2.4 and presented in Appendix A. The *examples* represent the material extracted from the literature used in qualitative analysis. The *explanations* summarize the example contents from which the CIPROS checklist was created.

Patient registries are a crucial part of medical research. High quality registries use efficient information systems software selected from a wide variety of existing software solutions.

An efficient selection process requires focused selection criteria. The evidence-based CIPROS checklist [2] accelerates this requirements engineering process.

CIPROS was developed in a multistep procedure: (1) A systematic literature review provided an exhaustive collection of relevant publications (64 articles), (2) a catalogue of relevant criteria was derived by a qualitative content analysis, and (3) the checklist containing 72 items was composed which provides a minimal appraisal standard.

The data presented per checklist item provide the relevant textual information (examples) and a first qualitative summary (explanation). The examples and explanations provide the background information on CIPROS. They elucidate how to implement the checklist items in other projects. The literature list and the selected texts serve as a reference for scientists and system developers.

**Specifications Table**

**Table:** 

Subject area	*Medical informatics*
More specific subject area	*Patient Registry Software Systems*
Type of data	*Examples and explanations from which the CIPROS Checklist items are derived and which elaborate the CIPROS Checklist items demonstrative and detailed.*
How data was acquired	*Examples are chosen from the literature, explanations are created by the authors.*
Data format	*Text*
Experimental factors	*Examples are cited from reference papers, explanations are created by the authors.*
Experimental features	*Examples and explanations may inspire scientists and system developers how to implement the CIPROS checklist items in own projects. Examples and explanations of each Item of the CIPROS checklist can serve as reference book for scientists and system developers how to implement the items in own projects and systems.*
Data source location	*Examples are cited from the reference papers. Explanations are created by the authors. Reference papers are cited.*
Data accessibility	*The data are part of this article they are presented in*Appendix A*. We linked the respective CIPROS checklist Items directly to the examples and explanations in the elaboration part. This is a perfect way to make the elaboration part of the CIPROS checklist items directly accessible to the readers in a comfortable way.*

**Value of the data**

• A collection of references and examples how patient registries are implemented and used in the medical research community.
• Examples and explanations represent a wide range of practices, and provide the practical background how to implement CIPROS items in projects.
• The examples provide the raw data used to derive the CIPROS checklist.
• The structured collection of examples supports decision makers and developers to formulate project requirements and explain how to implement CIPROS.
• The data presented are a reference book for scientists and system developers on how to implement the features in their own projects.

## 1 Data

CIPROS is the result of a qualitative text analysis. This paper presents the qualitative data used for this process. It was created via a systematic review (see Section 2.1) which identified 64 relevant publications. These were read and qualitative information on relevant aspects was extracted. Therefore the data consists of citations from the analyzed papers (referred to as examples) and represent information describing individual aspects of systems and software structures for patient registries. From the text analysis we identified 72 relevant items and the corresponding data is shown. The data also contains qualitative summaries (called explanations) based on the citations per item. The text summaries were used to formulate the item content.

## 2 Experimental design, materials and methods

For the development of the CIPROS checklist we used qualitative content analysis (QCA) methods developed by Mayring [3] for social research, and adapted them to create a checklist which supports requirements extraction for patient registry software systems. To create the CIPROS checklist we performed three steps which are described below:

1) A systematic literature review
2) A qualitative content analysis
3) Creation of an early version of the checklist

### 2.1 Systematic literature review

A systematic literature review was performed in PubMed to find papers on patient registry software systems. This guarantees that the software systems are used in real-life medical research projects. The following search in PubMed was performed at 17th of January 2014: *“(registry or registries) AND (eCRF or EDC or CDMS or CTMS or web) AND (software or open-source or open source or Java)”.* It was updated at 15st of February 2015.

The search terms were chosen in an iterative process: We are interested in registries with electronically Case Report Forms (eCRFs) or Electronic Data Capture (EDC) technology or Clinical Data Management Systems (CDMS) or Clinical Trial Management Systems (CTMS). We were mainly interested in software systems or open-source systems. We also included Java in our search terms.

### 2.2 Qualitative content-analysis

A qualitative content analysis according to Mayring [3] was performed. In a first step a process of inductive category development [3] was performed. We looked at titles and abstracts if there was a description of software related features. We looked at papers published in English or German. For respective papers, the full-texts were analyzed. Papers were considered for further analysis if a system description or at least a short system description was provided. We searched the papers for passages describing relevant software-specific features. We extracted these phrases describing software systems and built inductive categories out of the material, categorized the items or formulated new categories. After reading about 10–50% of the papers we revised the categories. Then we read the remaining papers to find additional categories for system's features.

### 2.3 Creation of an early version of the item list for the CIPROS checklist

To create the checklist we performed the step model of deductive category application described by Mayring [3]. Compiling the phrases retrieved from the qualitative content analysis we created a summary of content and defined the items. Within a feedback loop we revised these categories and eventually reduced them to main categories. If we found a new feature, we added a new item within the respective category. A new item was added for each new feature, regardless how often we found the feature. We enhanced the checklist with items which we found in none of the papers, but we considered as important based on our own experiences.

The CIPROS checklist consists of 72 items organized within 12 topics which relate to features in system components (S), functional aspects (F), or design steps (D).

### 2.4 Explanation and elaboration of the CIPROS checklist items

The data are presented in Appendix A. The CIPROS Checklist items are linked to the respective elaboration data, the examples and explanations.

For some items, we do not provide a reference and cannot provide an example, as these items were not discussed in the literature that we found in our SLR. However, they are considered relevant based on our own experience and theoretical reasoning (items no. 6.10, 6.12, 6.15, 9.2).

## Footnotes

Appendix A Supplementary data associated with this article can be found in the online version at http://dx.doi.org/10.1016/j.dib.2017.07.075.
