# Supplementary material for: Data for the elaboration of the CIPROS checklist with items for a patient registry software system: Examples and explanations
Source: Data Brief. 2017 Aug 3;14:494–7. doi: 10.1016/j.dib.2017.07.075 (PMC5558619; doi:10.1016/j.dib.2017.07.075)
Supplement: Supplementary file 2 — Supplementary material [file mmc2.pdf]

Appendix A: The Checklist with Items for Patient Registry Software Systems (CIPROS) linked with the related data, examples and explanations for the elaboration of the CIPROS Checklist items.

Table 1. CIPROS - A Checklist of Items to consider when choosing or developing a software system for patient registries. [Strg+Clicking on the Item No. will jump to the respective elaboration part of the Checklist Item.]

Aspect / Topic	Item No.	Description	Relevant References
**Software architecture**	**1**	**This topic contains items related to the architecture of the patient registry software system.**	
System Architecture	**1.1**	The system has a modular multi-tier architecture.	[1], [2], [3], [4], [5]
Platform independence	**1.2**	The system runs on different platforms.	[6], [7]
Open Source	**1.3**	Open source components are used to create the software of the patient registry system and it is made open source.	[8]
**Development**	**2**	**This topic comprises aspects which are important during the development process of a new registry project.**	
Design model	**2.1**	The system itself is developed following a design model.	[9]
Framework-based design	**2.2**	The system provides a framework for the development process of a new registry project.	[1], [5]
Questionnaire builder	**2.3**	The system has a table-based or web-based questionnaire builder.	[1], [9]
Usability testing	**2.4**	Usability of the system is tested. Users get involved in the development process early.	[9], [10], [11]
Performance testing	**2.5**	The performance of the system is tested.	[12]
**Interfaces and Interoperability**	**3**	**This topic comprehends different kinds of interfaces of the registry software system and related aspects of these interfaces.**	
**End-User Interfaces**		**This subtopic contains interfaces to end-users and related aspects.**	
Web Interface	**3.1**	The patient registry software system has a web interface.	[4], [6], [11]
Compatibility	**3.2**	The web interface is compatible with the major web browsers.	[4]
Email-alert	**3.3**	Email alerts are possible, as reminders for follow-up, etc.	[15]

Aspect / Topic	Item No.	Description	Relevant References
Messaging interface	**3.4**	There is a messaging interface to provide information for the end-users.	[1]
Online discussion forum	**3.5**	An online discussion forum for the end-users is established.	[1]
Mobile interface	**3.6**	An interface for mobile devices is available.	[1], [4], [16]
Patient interface	**3.7**	The system provides also a patient interface, where quality of life (QOL) and other information can be collected.	[9]
**Programming interface**		**This subtopic describes programming interfaces to other patient registries and related aspects.**	
Third party access	**3.8**	Programmatic access to data from an external resource is possible.	[1], [4]
API for inserting data	**3.9**	The system provides an application programming interface (API) for inserting data automatically.	[1], [4], [17], [18]
API for retrieving data	**3.10**	The system provides an API for retrieving data.	[4], [9], [18]
Data update mechanism	**3.11**	There is an update mechanism for automatically inserted data.	[5]
**Interfaces to other systems**		**This subtopic contains Interfaces to other systems.**	
Interface to HIS / CIS	**3.12**	An interface to HIS / CIS[1] (HL7)[2] is available to exchange data.	[9], [17]
Integration of biological data	**3.13**	The system has an interface to integrate pseudonymized biological data (see item 6.1). See for example the concepts of the TMF e.V. [19] or an anonymizing tool like described in Prasser et al. [20].	[4]
Extensibility is possible	**3.14**	It is possible, if necessary, to create further interfaces to other systems.	[5]
**Interoperability and Semantics & Standardization**	**4**	**This topic contains issues of interoperability with focus on semantic and standardization aspects.**	**[21], [22]**
CRFs	**4.1**	Standardized CRFs are used whenever possible	[1], [4]
Data	**4.2**	Standardized Data are used whenever possible.	[4]
Metadata	**4.3**	Ontology-based, standardized metadata are used.	[4], [17]
Vocabularies	**4.4**	Ontology-based, standardized vocabularies are used.	[4], [8]

[1] Hospital Information System (HIS) / Clinical Information System (CIS)
[2] Health Level Seven (HL7), http://www.hl7.org/

Aspect / Topic	Item No.	Description	Relevant References
XML Schema	**4.5**	An XML[3] schema definition (XSD) is available for structured data exchange.	[1], [2]
**Internationality**	**5**	**This topic contains an item which a registry software system can provide to support international cooperation in a registry project.**	
Multilingualism	**5.1**	The whole questionnaire life-cycle can be displayed in different languages.	[9], [15]
**Data management, data quality and usability**	**6**	**This topic contains important items which should be considered by the data management to support data quality and usability of the registry.**	
Pseudonymous patient identifier	**6.1**	A pseudonymous patient identifier (PID) is created by the system.	[4], [23]
CRF is divided in parts	**6.2**	The CRF is divided in logical parts.	[4]
Customizable CRF parts	**6.3**	CRFs are customizable according to the user's selections.	[1]
Minimal and extended dataset	**6.4**	If requested, a minimal and extended dataset can be used.	[11]
All data types are supported	**6.5**	The system supports the use of all common data types.	[5]
Special data types are possible	**6.6**	Special data types, like images, X-rays, and links can also be stored.	[5]
Multiple choice is used	**6.7**	Multiple choice data collection is used whenever possible.	[15], [24]
No predefined selection	**6.8**	No predefined selection is used, to avoid unwanted entries.	[10]
Data validation components	**6.9**	The system has implemented data validation components (Hard- and Soft Checks).	[1], [4], [11], [18], [25]
Data query tool	**6.10**	The system has a query tool to perform automatic data queries.	
Interface for manual data check	**6.11**	The system has an interface for manual data check.	[26]
Manual data	**6.12**	It is possible to perform manual data queries within the system.	

[3] Extensible Markup Language (XML), https://www.w3.org/XML/

Aspect / Topic	Item No.	Description	Relevant References
queries			
Data Query Flags	**6.13**	Entries with unresolved queries are marked with flags at different levels (item, part, patient, etc.).	[23]
Plausibility Flags	**6.14**	Implausible entries are marked with flags at different levels.	[25]
Insertion of unplanned visits	**6.15**	Unplanned visits can flexibly be integrated.	
Software ergonomics	**6.16**	The system should be designed following the standards of software ergonomics, defined in ISO 9241-110 [27].	[9]
**Data Analysis**	**7**	**This topic contains issues with which the registry software system can support data analysis.**	
Query builder for researchers	**7.1**	The system has a query builder to assist researchers to select interesting patient cohorts.	[8]
Report generation	**7.2**	The system is able to generate reports for selected cohorts.	[8]
Download of datasets	**7.3**	Datasets can be generated and downloaded for analysis in different formats, complete or for selected cohorts.	[6], [8], [10]
Graphical presentation of results	**7.4**	Results can be presented as colored graphs in real time.	[8], [9]
Risk Analysis	**7.5**	The system gives interactive feedback, classifying the patient through to implemented knowledge bases or scoring systems.	[9], [16], [23]
**Security aspects**	**8**	**This topic contains security aspects of the registry software system and important security aspects of the registry operation process.**	
Authorized users	**8.1**	Only authorized users have access to the data.	[1], [4]
Role-based access	**8.2**	The system provides role-based user access.	[4], [11]
Encrypted data transfer	**8.3**	The system utilizes secure web server communication through encrypted data transfer.	[1], [4], [6], [11]
Encrypted data storage	**8.4**	Sensitive data can be stored encrypted in the database.	[1], [4]
Audit trail	**8.5**	All changes in the database are tracked and monitored through an audit trail.	[4], [5], [15], [25]
Master-Slave replication	**8.6**	If necessary, master-slave replication should be established.	[1]
Backup management	**8.7**	Backups are stored separately regularly.	[1]
Firewall	**8.8**	The server is behind a firewall.	[26]

Aspect / Topic	Item No.	Description	Relevant References
Server room	**8.9**	The server room is locked and temperature controlled.	[3], [4]
**Privacy**	**9**	**This topic describes privacy aspects of the registry and of the software system additional to items 6.1 and 11.4.**	
Data Protection concept	**9.1**	A data protection concept should be established before starting a registry project.	[11]
Double pseudonymization	**9.2**	The registry software system should provide double pseudonymization for biological and genomic data.	
**General Features**	**10**	**This topic contains general items which have no direct relation to the other topics.**	
Costs	**10.1**	The costs of a patient registry software system must be taken into consideration, when choosing a software system: Is there a realistic calculation of the costs considering the costs of the procurement / programming, operation, life-cycle, archive, system replacement? Are the financial resources sufficient regarding the anticipated costs of sustainability of the registry guaranteed?	[28], [29]
Multi-client capability	**10.2**	The system has a multi-client capability. Several projects can simultaneously be executed in one installation of the system, but should be strictly separated.	[8]
Update mechanism	**10.3**	An update mechanism for the system is in place.	[9], [13]
Source documentation in pdf	**10.4**	Source documentation of CRFs in pdf format is possible.	[12]
**Organizational**	**11**	**This topic comprises software-related organizational items.**	
Compliance with regulations	**11.1**	The system is compliant with all known relevant regulations.	[11]
Informed Consent	**11.2**	The registry is compliant with Chapter 11, Title 21, Code of Federal Regulations and HIPPA.	[4], [28]
Rights on the data	**11.3**	There are clearly defined rules which describe the rights on the data for each participating institution.	[4], [6], [11]
Data protection guidelines	**11.4**	The system is compliant with all known appropriate data protection guidelines of the registry project.	[11]
**Training**	**12**	**This topic contains items which are important for the user training.**	
User manuals	**12.1**	There should be manuals for the registry end-users and for the operators.	[4], [12]

Aspect / Topic	Item No.	Description	Relevant References
User training	**12.2**	At the beginning, and if necessary during the project time, a training is provided for the users.	[4], [17]
User feedback	**12.3**	Regularly user feedback is collected for further improvements of the system.	[4], [30], [31]
Online help	**12.4**	The system provides an online help for data entry.	[10]

Explanation and Elaboration of the CIPROS Checklist Items

1. **Software Architecture (S)**
   This topic contains items related to the architecture of the patient registry software system.

Item 1.1 The system has a modular multi-tier architecture.

Examples

> "The CNRDS is implemented using a JavaEE three-tier, web-based architecture: 1) a web interface that interacts with the user, 2) a middle tier that contains the application's business logic, and 3) an integration tier that consists of the enterprise resources. The CNRDS runs on an Apache Tomcat web server and has a MySQL cluster as the back-end database." [1]

> "The architecture of CEMARA is based on a n-tier architecture. Via a web browser, the client tier connects to the middle tier, which is connected to several databases: the production database, the geographical dictionary database, and the thesaurus database. A data warehouse and a geographical information system allowing queries and representation are in progress. Their framework is close to an already available application for end-stage renal disease. The middle tier supports client services through Web containers and business logic services through component containers. Business logic in the middleware is interfaced with a SGBD dependant handler which supports the transactions toward the database. At the client side, CEMARA relies on existing local Internet networking facilities and on a widely spread computer configuration in medical settings. Access via a personal digital assistant is also available." [2]

> "The PCCR has been implemented using a three-tier, Web-based architecture: i) a client that interacts with the user; ii) an application server that contains the business logic of the application; and iii) a resource manager that stores the data. The PCCR utilizes Java Servlet/JSP technology and has Oracle 10g database as a back-end." [3]

> "The BCCR is a multi-tier web application that utilizes Java Servlet/JSP technology and has an Oracle 11g database as a back-end." [4]

> "So far, we have defined six optional modules, but the modular structure is easily extensible to fit on other needs. Out of those, five have already been implemented." [5]

Explanation

The system should be implemented as a multi-tier architecture or at least three-tier architecture to separate the business-logic from the database at the backend and the web server at the frontend [1], [2], [3], [4]. The data should be stored in one or more relational databases. The system should be

modular and extendable as the system presented by Deserno et al. [5] so that additional components and further developments can easily be integrated.

Item 1.2 The system runs on different platforms.

Examples

> "The FRB! software is a web-based application, which was developed using freely available software such as Apache, MySQL, PHP, and RubyonRails. This approach allows the application to be run on different server operating systems. Any device with Internet access and a recent browser can be used to interact with the application." [6]

> "**Database architecture**
>
> CDKD was designed as client–server architecture with PHP5 and MySQL, running on an Apache server. PHP, MySQL and Apache technology were preferred as they are open-source components and platform independent. CDKD is hosted on both windows and Linux platform." [7]

Explanation

In some cases it might be an advantage if the software runs on different platforms. If it is planned to run the system on different platforms or if it is not clear if this feature will be needed in the future, this item should be considered.

Item 1.3 Open source components are used to create the software of the patient registry system and it is also made available as open source.

Example

> "**Codebase**
>
> The i2b2-SSR code is available as open source software, licensed under LGPL version 3 for i2b2-SSR components; constituent components and dependencies are available under their respective open source licenses (repository and links at https://open.med.harvard.edu/display/CARRANET). The Webserver package is available from Cincinnati Children's Hospital Medical Center at https://bmi.cchmc.org/svn/i2b2/i2b2/public/." [8]

Explanation

To enable full interoperability beside semantics and standardization of metadata and vocabularies, open source components should be used to create the system and the code of the software system can be made open source as stated in the above example from Natter et al. [8]. The National Cancer Institute supports with the National Cancer Informatics Program [NCI NCIP] interoperability through providing source code, semantics and standardization of CRFs, vocabularies and metadata.

2. Development

This topic comprises aspects which are important during the development process of a new registry project.

Item 2.1 The system itself is developed following a design model.

Example

> "***Development process***

For this project Extreme Programming (XP) [28] has been adopted as software development approach. XP is considered a flexible approach to planning, making it well suited to react on changed requirements during the course of the project. As suggested in XP, the CHES development team adopted an incremental software architecture to match the demands of the various user groups. More specifically, the software architecture was not fixed upfront, but rather evolved over time. For this purpose, periodic releases and resulting feedback allowed for driving the development of CHES in a direction, which ensured the acceptance in daily clinical routine." [9]

Explanation

The software of the patient registry system should be developed following a design model to avoid ad hoc implementation and unstructured extensions.

Item 2.2 The system provides a framework for the developmental process of a new registry project.

Examples

"Nephrologists from the Registry Board of Advisors participated in the development of the CRFs in an effort to delineate the current standard of care of the patients with kidney disease and to facilitate consistent, thorough, and precise patient evaluations throughout the nephrology community. Nephrologists were given a Microsoft Excel spreadsheet for entering the required metadata information (e.g., field name, data type, value range, and presentation style) about each measurement in each case report form. An IT support team staff collected worksheets via electronic mail and then built web-based electronic forms for data collection. A hyperlink to the prototype application was given to the nephrologists along with instructions for testing and further iteration of the metadata spreadsheet. When feedback returned, the IT support team edited the electronic forms and republished them. The CNRDS utilizes CapitalBio electronic medical records (EMR) system's (CapitalBio Corporation, Beijing, China) dynamic case report form (dCRF) technology to enable modification and publishing of the CRFs on-the-fly. The case report forms created from the CNRDS are presented in Table 1. Additionally, the following administrative data elements are stored along with the CRFs: date when the case was submitted, registering institution code, and the person who submitted the data." [1]

"As parts of the core functionality of any RDR, we suggest five modules: Data Core, Access Control, Audit Trail, EDC, and Term. All data is stored in a relational database, but—according to the special needs of any instantiation of such a registry—the data tables, their fields, and the respective web rendering may vary." [5]

Explanation

The system should provide a framework for the development of a new registry project, for example as described by Deserno et al. [5] through specific modules. There should also be a framework for building up the project-specific content. This minimizes the need for programming skills for persons developing for example the medical content such as questionnaires, variables, metadata, range values for variables and variable coding.

Item 2.3 The system has a table-based or web-based questionnaire builder.

Examples

"Nephrologists were given a Microsoft Excel spreadsheet for entering the required metadata information (e.g., field name, data type, value range, and presentation style) about each measurement in each case report form. An IT support team staff collected worksheets via electronic mail and then built web-based electronic forms for data collection. A hyperlink to the prototype application was given to the nephrologists along with instructions for testing and further iteration of the metadata spreadsheet. When feedback returned, the IT support team edited the electronic forms and republished them. The CNRDS utilizes CapitalBio electronic medical records (EMR) system's (CapitalBio Corporation, Beijing, China) dynamic case report form (dCRF) technology to enable modification and publishing of the CRFs on-the-fly." [1]

"Questionnaire Builder: CHES Questionnaire Builder was developed for defining the structural properties (i.e., question and answer texts, or psychometric item characteristics) of questionnaires. In addition, CHES Questionnaire Builder enables researchers to define the visual appearance of questionnaires in order to adapt them to different devices, e.g., tablets, smart phones, or specific patient groups (e.g., elderly people, visually impaired)." [9]

Explanation

For the developmental process a questionnaire builder should be available, then this part can be done by persons who are more familiar with the content of the planned project and must not be done by the system developer. This reduces communication time and misunderstandings.

Item 2.4 Usability of the system is tested. Users are involved in the developmental process early on.

Examples

"Usability prototyping and usability testing ensure that users are included at an early stage (standards for user oriented software are specified in ISO 13407). It is crucial to tailor the software to the specific needs and abilities of potential users. We identified several user groups for CHES leading to specific requirements on the availability of certain features for each of them: …" [9]

"The first precondition to acquire good quality data is to pay a special attention to the GUI design. Unfortunately, physicians are not much interested in the design phase, and quite often, they let computer scientists and technicians decide for them. Being end users, physicians simply request easy and fast data entry procedures, and it is also not uncommon that they ask to replicate the same layout of their previous paper-based clinical charts. Nevertheless, a careful and shared preliminary analysis is mandatory to anticipate as much as possible any type of future exploitation of the acquired data. In fact, the impossibility to accomplish the necessary statistics is usually realized at a later time when it's too late for revising the model." [10]

"To ensure intuitive usage of the registry application, data entry staff members such as study nurses have been involved in its development at an early stage." [11]

Explanation

The usability of the system should be tested and therefore end-users must be involved in the developmental process at an early stage. This is crucial to avoid insertion errors and adding missing questions. It is also necessary to sensitize users for the content, and that all data which are required for the analysis will be captured.

Item 2.5 The performance of the system is tested.

Example

"*Performance results*

The performance was evaluated by emulating 500 simultaneous users interacting with the web application. We evaluated the performance by measuring the load times of the web frontend (as shown in Figure 6). Each user calls the website through an http request. The evaluation was performed on a client that has an Intel Core 2 Duo CPU with 2.4 GHz and 4 GB RAM, running the 64-bit Windows 7 OS, Java Runtime 1.6.21 and Apache JMeter. The web application was deployed on a Windows XP Pro OS server on an AMD Athlon 64 X2 Dual Core Processor 5200+ with 2.7 GHz and 3 GB RAM. The server has the Glassfish 3.0 Application Server. It is expected that initially only 10–50 users will access the application. This corresponds to a load time between 28 and 48 ms, which results in an excellent quality of experience of the users. Figure 7 shows the throughput and number of kilobytes per second for 10–500 users. Table I shows the detailed results of a typical user scenario, measured by FireBug, a plug-in for web browser Firefox. The user logs in on the website with his/her user credentials, downloads an empty PDF questionnaire, fills in this template and uploads the form. Later, the user reviews and analyses these data on the website. The display and download times are within the acceptable range,

but the PDF takes more time due to the generation process. In addition, the execution times for the retrieval of data analysis cases were also measured. Figure 8 shows the execution times for the audiological and neurological data for several data analysis cases. One case had a longer execution time and took 61.12 s. All other cases' execution time ranges from 0.23 to 2.54 s and are shown in Figure 8. The queries for audiological data take more time due to a higher number of variables in the queries." [12]

Table I. Website performance measurements.

Webpage	Size(kb	Time (s)	Std dev. (s)
Login page	613.7	4.036	0.957
Physician overview	623.4	3.319	1.031
Download page	624.9	3.998	1.277
PDF selection page (registr)	291.2	12.950	1.321
PDF selection page (follow)	283.2	11.924	1.421
Patient list page	670.8	5.534	1.853
Data analysis page	807.7	6.564	1.515

Explanation

The complete system architecture of a new registry project should be planned, taking into account the number of end-users who will work simultaneously with it. Before starting a new project, the performance of the patient registry software system should be tested to prevent system failures caused by system overload.

3. Interfaces and Interoperability (S)

This topic comprehends different kinds of interfaces of the registry software system and related aspects of these interfaces.

End-User Interfaces

This subtopic contains interfaces to end-users and related aspects.

Item 3.1 The patient registry software system has a web interface.

Examples

"The BCCR public website (Fig. 2) can be accessed at http://bccr.unmc.edu/." [4]

"There are two ways of entering data into the FRB! system: via a web interface or via a third-party software interface. If the User has no electronic patient management system, the independent web-based application, which can be accessed from a wide range of devices and operating systems (e.g., Windows-PC, Macintosh, tablet computer, mobile phone), can be used with a regular browser (e.g., Safari, Internet Explorer, Firefox, Opera). Additional software on the user's terminal is not required." [6]

"The CERTAIN web application, accessible via http://www.certain-registry.eu/RegApp, supports, therefore, not only the data entry, presentation, visualization, and export, but also the automatic and manual data validation. These functionalities are active at any time and location, requiring only a common web browser and internet access." [11]

Explanation

We recommend a web interface for patient registry software systems. Web-based data entry is a very comfortable and state of the art way to collect patient data for multi-center registries. All data is

immediately available in the database. The data from all centers are collected in the same way, no data-integration with the data from different centers is necessary. No additional software must be installed on the client side. When a software update is necessary it must only be installed on the server, the clients get a new version when they log in next time.

We recommend a real web-based system which requires no additional software-installation at the client-side. Compared to Lycett et al. [13] and Wake et al. [14] in their registry project software installation turned out as complicated, time consuming and prevented some groups from participating in the registry project since they failed to install the required software.

Item 3.2 The web interface is compatible with the major web browsers.

Example

> "The BCCR user interface is compatible with all major web browsers, such as Microsoft Internet Explorer 6+, Mozilla Firefox 3+, Opera, and Safari." [4]

Explanation

The system should be compatible with all common web browsers, such as Microsoft Internet Explorer, Mozilla Firefox, Sea Monkey and Safari, that most of the clients can use their familiar web browser.

Item 3.3 Email alerts are possible as reminders for follow-up, etc.

Example

> "For investigators, automated email reminders notifying follow-up times for individual patients will be sent two months before the evaluation is due. If participating families wish to opt-out of the study, completion of an End of Study form will be required.
>
> For parents/caregivers who have elected and agreed on the patient informed consent form to respond directly on-line, an automated email reminder will be sent two months prior to the next follow-up evaluation for their child, and on the day of the scheduled follow-up. If a parent/caregiver do not respond and complete the recommended evaluations for a particular time-point, the clinician will be subsequently notified by email. A notification will be sent to the clinician, once the parent has completed their data entry for that time-point." [15]

Explanation

It should be possible to send emails from the system to the end-users, mainly to send them administrative information about their usernames, passwords, etc. but also for patient management for example as reminder for follow-ups.

Item 3.4 There is a messaging interface to provide information for the end-users.

Example

> "**Online discussion forum**
>
> … The CSN uses this forum to make announcements and publish SOP documents and online surveys. …
>
> **Social networking**
>
> … We introduced two social networking features: online discussion and messaging." [1]

Explanation

The system should provide a messaging interface to provide information like SOPs, user manuals, etc., and to make announcements to the end-users.

Item 3.5 An online discussion forum for the end-users is established.

Example

> "**Online discussion forum**
>
> The web forum embedded within the CNRDS promotes active discussion among the renal community. People in China are familiar with online discussion forums. The professional online forum for kidney disease can facilitate interactions between nephrologists, including experts in the field. They use the platform to ask questions and share their medical experience and expertise. Feedback and suggestions for the CNRDS are also posted on the forum. The CSN uses this forum to make announcements and publish SOP documents and online surveys. From April 22nd, 2010 to Nov 15th, 2011, 2,441 threads composed of 9500 messages were exchanged on the forum. Table 4 shows a summary of topics and threads. At the first couple of months when the CNRDS just launched, physicians are not familiar with it, most of the posts are about how to use the system. After that, topics are more and more concentrated in the discussion of dialysis. Weekly digest of the forum posts are reported to the Board of the CSN. The online forum has been an important communication channel for nephrologists and had a positive influence on patient care." [1]

Explanation

The patient registry software system should provide an online discussion forum, to enable active discussion and interaction for the participants. As described by Xie et al. [1] in the above example, this can have a positive influence on patient care.

Item 3.6 An interface for mobile devices is available.

Examples

> "The latest generation of smartphones, such as Apple's iPhone and various Google's Android touch screen devices, are increasingly viewed as handheld computers rather than phones, due to their powerful on-board computing capability, capacious memories, large screens and open operating systems that encourage application development [30]. It is clear that the potential for mobile communication to transform healthcare and clinical intervention in the community is tremendous. Several previous studies have evaluated the use of mobile phones to support healthcare and public health interventions, notably in the collection and collation of data for healthcare research and education purposes [31,32]. Rather than developing native apps for different platforms, we built the CNRDS mobile interface using HTML5 and the jQuery mobile framework [33]. The mobile version works well on the iPhone (see Figure 3), iPad, and Android phones." [1]
>
> "To improve portability, convenience and ease of use, a separate interface for Apple's iPads has been created." [4]
>
> "The eTHR was designed as a downloadable, multiplatform, web-based application, initially for use on the iPad (Apple Computers). The user interface was built using the jQuery Mobile 1.0 Framework and was designed to save and update data in a MySQL database through asynchronous javascript requests sent over Secure Socket Layer (SSL) encryption. Data transfer, with 128-bit encryption, was designed to enable synchronous or intermittent upload of data to dedicated, secured servers within a host site." [16]

Explanation

If necessary and useful for the project an interface for mobile devices should be available, to enable data entry from distributed locations.

Especially in rural, under-resourced environments the use of mobile devices becomes a necessary tool to manage information for patient care effectively Zargaran et al. [16]. An interface to mobile

devices can help to collect distributed data and integrate them in a patient registry. Therefore a patient registry software system should provide this interface.

Item 3.7 The system also provides a patient interface where quality of life and other information can be collected.

Example

> “***Implementation***
>
> By 2012 CHES has been implemented in a number of hospital settings in various fields of medicine (e.g., urology, nephrology, orthopedics, radiation therapy, neurology, oncology, gynecology, health psychology) in Austria, Germany, Switzerland, and the UK. So far, about 5000 patients completed the computerized questionnaires with a total of approximately 15000 assessments. Its web-interface for completing questionnaires and case report forms online is currently used in an international EORTC questionnaire validation study (EORTC QLQ-TC26) in five European countries and Australia, and in a CAT validation study in Denmark.
>
> At these centers data assessment is done via tablet- PCs with screen sizes of 10" or 12". Usually one to four questions depending on screen size and patient’s eyesight are shown at once. In most settings, a client–server solution for data storage is in place based on either Wi-Fi or LAN. This is necessary in order to provide results without time loss at all working stations required.” [9]

Explanation

If appropriate within the project a patient interface should be provided. Some data, such as quality of life (QOL) data, can be inserted by the patients themselves. If a patient interface is provided, it is useful to provide an interface for mobile devices (Item 3.6) to collect these data as well.

Programming Interface

This subtopic describes programming interfaces to other patient registries and related aspects.

Item 3.8 Programmatic access to data from an external resource is possible.

Examples

> “**The CNRDS application programming interface (API)**
>
> Prior to the nationwide CNRDS, Shanghai, Beijing, and Zhejiang provinces established their own local renal registries. Indeed, these three registries have been running for several years and contain valuable data. In order to maximize the value of the previous investment, a RESTful web service API was developed for third party software providers to exchange their data with CNRDS. The RESTful web service was chosen due to its streaming capability and on-the-fly compression [35]. The data exchange format is a customized XML schema, which is downloadable as an additional file of this manuscript (Additional file 1). Each regional registry was assigned a unique API key for discrimination. Because the API is an interface between machines, third party software vendors should ensure that the data they submit is validated and complete. Data validation rules are also performed on the CNRDS server; an error code will return if the submission contains abnormal data.” [1]

> “**Programming interface**
>
> BCCR end-users utilize a web interface for data entry and management. To allow third-party applications to access the BCCR’s data directly and to satisfy the caBIG® bronze compatibility requirements, a set of application programming interfaces (API) has been developed.” [4]

Explanation

If necessary within the project, the patient registry software system should provide an Application Programming Interface (API) to enable participants to insert and retrieve data automatically in the system. This may be an important tool for data interchange and cooperation within different institutions.

Item 3.9 The system provides an application programming interface (API) for inserting data automatically.

Examples

"Because several local dialysis registries have been established [5,19,20] prior to the national one, the RESTful web service can be used as a data exchange interface for automatically importing data from third party registries." [1]

"This set of APIs consists of methods for both retrieving data from and inserting data into the BCCR." [4]

"In order to avoid duplicate data entry, the EHR from the Clinics Hospital of the University of Saõ Paulo Medical School (HCFMUSP) was integrated to the EDC through the REDCap API (Application Program Interface). The REDCap API is an interface that allows external applications to connect to REDCap remotely, and it is used for programmatically retrieving or modifying data or settings within REDCap. As the API is a built-in feature of REDCap, no installation is required and this tool implements the use of tokens as a means of authenticating and validating all API requests that are received. In addition, the API also implements data validation when the API is used for data import purposes in order to ensure that only valid data will be stored. By using the REDCap API, it was possible to retrieve useful demographic information directly from the sources of hospital systems." [17]

"Data were collected using handheld Samsung (Seoul, South Korea) Galaxy Tabs with a 7-inch display running Android operating system; 50 tablets were used during the census, and 40 during the vaccination campaign. The software platform was built by a contracted partner (Majella Global Technologies, Portland, ME, USA) on Open Data Kit. External battery packs with dual USB charging ports provided a portable, backup power supply. Data records were first stored locally on devices in the field, and uploaded nightly via office Wi-Fi to a secure, web-hosted database. …

Each night, data records collected in the field were uploaded from each tablet and merged into a web-hosted database." [18]

Explanation

Inserting data automatically from other registries is useful for registry projects which deal with retrospective data, or when data is captured in systems other than the registry. It enables cooperation between different institutions and prevents errors which occur if the data were inserted twice by hand. It is an innovative way to enable cooperation within different registries and it supersedes the cumbersome exchange of data, for example with excel sheets, and then imported using individual programs.

Item 3.10 The system provides an API for retrieving data.

Examples

"This set of APIs consists of methods for both retrieving data from and inserting data into the BCCR." [4]

"Data Export/Import: Sociodemographic, clinical and questionnaire data can be exported to different file formats and imported from files, e.g., SPSS or MSExcel." [9]

"Online registries were subsequently downloaded nightly for analysis. …

At the end of census, population data were downloaded from the webhosted electronic database and formatted in Microsoft Excel to become a dataset, or a ''lookup table''. The lookup table was loaded

back on to all tablets and embedded within the electronic forms, and served as a locally stored database from which previously collected population data could be retrieved.“ [18]

Explanation

A tool fulfilling this item should be provided by all systems, it is necessary to provide data for analysis tools such as SAS or R, also to return data to the participating institutions.

Item 3.11 There is an update mechanism for automatically inserting data.

Example

“Basically, the BLOB module ensures that large data files are safely transferred via the internet and attached to the subject’s identifier providing the date of filing. The date of transfer and the transferring person’s identifier are logged in the audit trail module. Versioning of data is possible, since the module defines a document identifier (DID) that is not unique, and a Boolean flag “latest” indicating the latest version of the respective DID.” [5]

Explanation

If the system provides automatic data import from external resources it may be useful to update already inserted data regularly. This feature should also be available to a patient registry software system. Like in the above mentioned example from Deserno et al. [5] the update of binary large objects (BLOBs) is possible, so it should also be possible to update common data.

Interfaces to other systems

This subtopic contains interfaces to other systems.

Item 3.12 An Interface to HIS / CIS (HL7) is available to exchange data.

Examples

“Interface to clinical information systems (HL7): In order to exchange medical and sociodemographic data between CHES and clinical information systems (CIS) a HL7-interface is available.” [9]

“In order to avoid duplicate data entry, the EHR from the Clinics Hospital of the University of Saõ Paulo Medical School (HCFMUSP) was integrated to the EDC through the REDCap API (Application Program Interface). The REDCap API is an interface that allows external applications to connect to REDCap remotely, and it is used for programmatically retrieving or modifying data or settings within REDCap. As the API is a built-in feature of REDCap, no installation is required and this tool implements the use of tokens as a means of authenticating and validating all API requests that are received. In addition, the API also implements data validation when the API is used for data import purposes in order to ensure that only valid data will be stored. By using the REDCap API, it was possible to retrieve useful demographic information directly from the sources of hospital systems.” [17]

Explanation

The system should provide an interface to Clinical Information Systems (CIS) to enable the automatic import of clinical data and reduce the error rate by entering data twice or transferring data manually from one to another system.

Item 3.13 The system has an interface to integrate pseudonymized biological data (see item 6.1). See for example the concepts of the TMF e.V. [19] or an anonymizing tool described by Prasser et al. [20].

Example

"**Integration with caTissue**

The caTissue Suite,31 which is the tissue bank repository tool developed under the caBIG® umbrella, has been adopted and integrated with the BCCR to collect and manage the biospecimen data in a standard and efficient way. It is used to track the collection, storage and distribution of specimens and provides quality assurance for all of these activities. The participating centers are able to either submit biospecimen data into the central repository or maintain their own installation of caTissue and store biospecimen data locally." [4]

Explanation

If it is planned to analyze biological data within the registry project the software system should provide a module for integrating biological data. Since personalized medicine is becoming more and more popular, we suggest that a patient registry software system should provide this feature. When integrating biological data they must be pseudonymized, see for example the concepts of the TMF e.V. [19] or an anonymizing tool described by Prasser et al. [20].

Item 3.14 Extensibility is possible

Example

"So far, we have defined six optional modules, but the modular structure is easily extensible to fit on other needs. Out of those, five have already been implemented." [5]

Explanation

As already mentioned in item 1.1, the architecture of the system should be extensible so new modules can easily be integrated if they turn out to be necessary during the project time. This is also valid for new interfaces. If it turns out during the project time that a patient interface or an interface for mobile devices would be helpful, it should be possible to integrate them in the system.

4. Interoperability and Semantics & Standardization (F)

This topic contains issues of interoperability with focus on semantic and standardization aspects.

Item 4.1 *Standardized CRFs are used whenever possible.*

Examples

"Data collection forms

To promote the acquisition of high quality, clinically meaningful data, standard data collection forms were defined according to the blood purification standard operation procedures (SOPs) developed by the CSN [21,22], which outline CKD-related signs, symptoms, laboratory tests, and treatments that are internationally accepted to monitor onset, progression, and outcomes over the lifelong course of the disease. Nephrologists from the Registry Board of Advisors participated in the development of the CRFs in an effort to delineate the current standard of care of the patients with kidney disease and to facilitate consistent, thorough, and precise patient evaluations throughout the nephrology community." [1]

"The BCCR questionnaires have been designed and developed to collect comprehensive data related to the diagnosis, treatment and follow-up of BC patients, as well as information pertaining to demographics and survivorship. Existing, well established and recognized in the cancer research community questionnaires, such as the SF-36v2 Health Survey to measure QOL[26] and the NCI Quick Food Scan questionnaire[27] for the dietary habits have been implemented in the BCCR registry. The American Cancer Society's (ACS) examples of moderate versus vigorous physical activity guidelines for cancer prevention[28] were used to create the physical activity form. Sleep habits are assessed by using the Pittsburgh Sleep Quality Index.[29]" [4]

Explanation

We suggest the use of standardized case report forms (CRFs), because the use of standardized CRFs supports a unified collection of research data and is a prerequisite for multi-center analyses at a later stage. In addition it simplifies data integration from multiple centers at a later stage.

Item 4.2 *Standardized Data are used whenever possible*

Examples

> "The data elements of the BCCR vocabulary have been defined based on the caDSR convention and, when possible, were mapped to the caDSR." [4]

Explanation

We propose to collect standardized data whenever possible, because they are comparable and it is much easier to analyze them.

Item 4.3 Ontology-based, standardized metadata are used.

Examples

> "BCCR data element descriptors (metadata) have been constructed from the aforementioned vocabularies using the NCI CBIIT Data Standards Registry and Repository (caDSR) common data elements convention[25] and are available in an electronic format." [4]

> "Our registry adopted all applicable data elements and definitions in accordance with ACC/AHA available published data standards, including those developed for Electrophysiology, Atrial Fibrillation, Acute Coronary Syndromes, Heart Failure, and Cardiac Imaging [29–33]. Other data sources included data elements from large device clinical trials and registries, such as CTOPP (Canadian Trial of Physiologic Pacing) [34], MOST (Mode Selection Trial in Sinus Node Dysfunction) [35], COMPANION (Comparison of Medical Therapy, Pacing, and Defibrillation in Heart Failure) [36], REVERSE (Resynchronization reVErses Remodeling in Systolic Left vEntricular Dysfunction) [37]. We also reviewed case report forms, data elements, and definitions from international data collection efforts. Examples of these data sources include the ACC National Cardiovascular Data Registry (NCDR) [38,39], Health Level Seven International (HL7) [40], Clinical Data Interchange Standards Consortium (CDISC) [41] and Cancer Data Standards Registry and Repository (caDSR) [42,43]. Finally, we also included standardized definitions for clinical endpoints and adverse events in cardiovascular trials from the US Food and Drug Administration (FDA) [44]." [17]

Explanation

We propose to use standardized metadata to support later cooperation with other centers and to support integration and multi-center analysis of the research data.

Item 4.4 Ontology-based, standardized vocabularies are used.

Examples

> "The following controlled terminologies have been implemented both in the BCCR front-end and metadata: NCI Thesaurus (NCIt)[23] and Systematized Nomenclature of Medicine-Clinical Terms (SNOMED-CT).[24] These publicly accessible controlled vocabularies meet all caBIG® Bronze requirements." [4]

> "Shared Ontology service (F)

> The Shared Ontology service provides web service access to hierarchical vocabularies that describe i2b2 data elements and provide term mappings for i2b2 query panels. This component functions identically to the standard i2b2 Ontology Cell for query panels, with the additional external policy requirement that at least one common vocabulary exists and is mapped at all data contributor nodes. In practice, as part

of the Shared Ontology model, we additionally provide the requisite i2b2 concept dimension rows as a Shared Ontology service public table; however, these additional term mappings may be equivalently implemented at the SHRINE adapter translation layer.25 We also utilize a new, streamlined Shared Ontology module that incorporates Apache Lucene search capabilities." [8]

Explanation

We propose to use standardized vocabularies to support cooperation with other centers and to support faster integration and analysis of multi-center research data.

Item 4.5 An Extensible Markup Language (XML) schema definition (XSD) is available for structured data exchange.

Examples

"**The CNRDS application programming interface (API)**

Prior to the nationwide CNRDS, Shanghai, Beijing, and Zhejiang provinces established their own local renal registries. Indeed, these three registries have been running for several years and contain valuable data. In order to maximize the value of the previous investment, a RESTful web service API was developed for third party software providers to exchange their data with CNRDS. The RESTful web service was chosen due to its streaming capability and on-the-fly compression [35]. The data exchange format is a customized XML schema, which is downloadable as an additional file of this manuscript (Additional file 1). Each regional registry was assigned a unique API key for discrimination. Because the API is an interface between machines, third party software vendors should ensure that the data they submit is validated and complete. Data validation rules are also performed on the CNRDS server; an error code will return if the submission contains abnormal data." [1]

"*Interoperability*

CEMARA was conceived in order to communicate with other sources of information: the use of XML, as an exchange format, permits a greater flexibility and better capacities to exchange data with other information systems such as French Medical Insurance system or Hospital Information systems. It also allows importation of former databases (Figure 2)." [2]

Explanation

An XML schema definition for structured data exchange should be available to support interoperability, to foster active data exchange and communication between different centers, and to enable cooperative research projects.

5. Internationality (F)

This topic contains an item which a registry software system can provide to support international cooperation in a registry project.

Item 5.1 The whole questionnaire life-cycle can be displayed in different languages.

Examples

"Multilingualism: The whole life-cycle — designing questionnaires, administering questionnaires and displaying the results to physicians — was designed to accommodate for the need of supporting different languages. Therefore, nurses are able to select the language for questionnaire administration for each patient individually. The software is currently available in English, German and Italian. Further translations are currently ongoing." [9]

"The Cochlear P-IROS electronic, web-based platform is currently available in five languages; English, Mandarin, Korean, Japanese and Russian." [15]

Explanation

A patient registry software system should support internationality by providing multilingualism for the whole life-cycle – if necessary. For most international projects it may be sufficient to provide the whole life-cycle in English, but in some cases it may be necessary to provide the whole life-cycle in other languages as well.

6. Data management, data quality and usability (F)

This topic contains important items which should be considered by data management to support data quality and usability of the registry.

Item 6.1 A pseudonymous patient identifier (PID) is created by the system.

Examples

"*Administrative data* include: (i) date when questionnaire is submitted; (ii) current status of the questionnaire; (iii) registering institution code; (iv) clinician's ID; and (v) subject's identification code—an automatically generated number that can be used to re-identify the subject when data are deidentified (as permitted by HIPAA regulations)." [4]

"To limit the impact of potential exposure or leakage of patient information, the JADE Program does not store any identifying patient information electronically. No name or national identity number is captured and a case specific code (ADF code) is generated for each enrolled patient. Only the physician has the information to identify the patient given a specific ADF code. These case sensitive codes are known to the patients and all report forms generated electronically or in paper form, are kept by the physicians or in the case records as appropriate." [23]

Explanation

It is not necessary to store the personal information of the patients such as names, addresses or social security numbers in the registry. For patient identification a pseudonymous patient identifier (PID) should be created by the system when a new patient is allocated for the first time. This PID is used in the tables of the relational database to identify the corresponding patient data. The personal patient information should be stored separately and can be identified through the PID, which must be marked there. (See also item 8.4 Encrypted data storage).

Item 6.2 CRF is divided in logical parts.

Example

"According to the BCCR rules, information on personal, demographic, lifestyle, physical activity, dietary habits, family history, women's health, genetics data, symptoms, QOL, and medical history may be provided by a subject; whereas medical information on diagnostic studies, pathology/staging, treatment, surgeries, biospecimens, and survival can be provided only by clinical personnel. The Core Data Set categories included in this registry are described below." [4]

Explanation

The collected data in the eCRF should be divided in logical parts, as described by Sherman et al. [4] in the above example. Registries often collect a big number of variables they can be structured for different aspects, which data belong together, who will insert them, who will elevate them.

Associated variables should be collected together. Not all variables are available at one moment. Data can be structured, for example in demographic data, laboratory data, quality of life (QOL) data, treatment data, etc.

Item 6.3 CRFs are customizable according to the user's selections.

Example

> "**Figure 2 Data collection form fragment.** The initial erythropoietin (EPO) data collection form is shown in (a). As shown in (b) and (c), the visible data elements change according to the user's selections" [1]

Explanation

To avoid ambiguity, CRFs should be customizable according to the user's selections if necessary. In a customizable CRF, for example gender-specific questions can then only be shown if applicable, and the not applicable questions can be faded-out. This is timesaving and prevents people to insert their own abbreviations, because they want to show that the questions are not applicable.

Item 6.4 If requested a minimal and extended dataset can be used.

Example

> "The dataset is divided into 2 sections: minimal and extended. The minimal dataset is mandatory for all participating centers, and only data fulfilling these minimal requirements will be incorporated into the research database. The extended dataset facilitates deeper insight into patients' treatment and supports the documentation of additional items that are partly predefined and that can partly be defined by the participating center itself." [11]

Explanation

If a minimal and extended dataset can be used, this allows the definition of the core variables mandatory for all cases or most wanted in the registry and more specific documentation in other cases. Especially in multi-center studies this feature is often asked for because different centers capture different variables and so each center can keep their specific variables in the registry.

Item 6.5 The system supports the use of all common data types.

Example

> "Electronic data capture (EDC): the core purpose of any registry is to collect medical data on subjects electronically. All of such data must be given a data type, which can be either numerical, date/time, or one or more items selected from a predefined list (terminology, cf. next item). Regarding an appropriate statistical assessment, unstructured text is disadvantageous and shall be avoided. Numerical items must have a unit and a reference interval for instantaneous plausibility checks. Of course, EDC is an inherent component of any registry." [5]

Explanation

A patient registry software system should support the use of all common data types which will be selected in CRFs, like integers, booleans, characters, floating-point numbers, alphanumerical strings, and also advanced types such as dates and multiple choice selection. This enables the collected data which are relevant for the research to be stored directly in the database.

Item 6.6 Special data types, like images, X-rays, and links can also be stored.

Example

"Basically, the BLOB module ensures that large data files are safely transferred via the internet and attached to the subject's identifier providing the date of filing. The date of transfer and the transferring person's identifier are logged in the audit trail module. Versioning of data is possible, since the module defines a document identifier (DID) that is not unique, and a Boolean flag "latest" indicating the latest version of the respective DID. Terminology is used to classify the types of data (e.g., photograph, ECG recording, scanned diagnostic letter) as well as the according file endings such as the portable document format (PDF), portable network graphics (PNG), or DCM for digital imaging and communication in medicine (DICOM) files. Technically, the BLOB module parses, extracts, and handles BLOB data received from a hypertext transfer protocol (HTTP) request object. The request object is built by the hypertext markup language (HTML) file upload object, according to the specification of the Internet Engineering Task Force (IETF) [25]. … In the German Calciphylaxis Registry, the RDR BLOB module allows web-based image integration. Photographs that have been taken on patient's bed site are uploaded and linked to the subject ID in the registry. All images are described by their recording date and the body region that is visualized in the image. For precise localization, the body part terminology has been defined according to the image retrieval in medical applications (IRMA) code for medical images, i.e., a mono-hierarchical multiaxial classification scheme [28]. … Figure 5 (left) visualizes the BLOB module integrated into the German Calciphylaxis Registry. An overview of images is displayed, which can be magnified on the user's selection. The list can be accessed by patient ID, recording date, body region, detailed position, left or right hand side, and any combination of those (filter bars on top)." [5]

Explanation

It should be possible to store special data types like images, x-rays, links, etc. in the registry. For some diseases these data contain important information which should be available beside other patient data so the information in these data is available for analysis and further treatment.

Item 6.7 Multiple choice data collection is used whenever possible.

Examples

"The vast majority of responses on all forms are multiple choice, check box, radio buttons or pull-down response options to facilitate entry and reduce entry error. Minimal free-text response fields are included." [15]

"To minimize manual entry by the end user, drop down boxes and check boxes were used for data entry." [24]

Explanation

Whenever possible the data in a patient registry software system should be captured systematically by using multiple choice selections, drop down menus or radio buttons, etc. Free text fields should be avoided or only used if the data cannot be captured systematically. If the data are captured systematically they can be analyzed easily, while it is extremely time-consuming to evaluate free text.

Item 6.8 No predefined selection is used to avoid unwanted entries.

Example

"Another frequent user complaint is that data entry is too time-consuming. A common solution to streamline data entry consists of pre-instantiating all items with informative default values (e.g., choosing the most frequent value as the default). However, in that case, users could pay less attention to the input fields and stay with the default values even when they are not the correct ones. In order to compensate for that bias, we decided to use the "missing value" as default." [10]

Explanation

We recommend utilizing no predefined selection in the data entry fields. As stated by Lanzola et al. [10] in the above example, if predefined selection is used, users may pay less attention to the input

fields and use the default values even when they are not correct. This leads to false values in the database and when analyzed may falsify the results.

Item 6.9 The system has implemented data validation components (Hard-and Soft Checks).

Examples

“Data validation

CNRDS is dedicated to ensuring that the data entered into the registry is accurate. Formatting and acceptable range information for each data element was provided in the spreadsheet after the experts defined the CRFs. The IT support team used this information to build logical evaluations for the data. The user’s input is validated both server-side and client-side. The validation components of the web interface prevent the users from entering erroneous information into the system. A JavaScript validator checks the user’s input against the validation rules before submitting the form. If a user does not enter data in a required field, the system displays error messages requesting this information prior to accepting the entry for submission to the registry. The system will not accept the data until the required fields are filled in. The same applies if the data entered is out of the expected range. For example, CNRDS recognizes the expected range for an individual’s total bilirubin as between 0 and 1000 µmol/L. A warning message displays if the data entered by the user is below or above the acceptable range. This gives the user a chance to review the information he/she entered and apply any necessary corrections before submitting the data. Similar algorithms are performed server-side before the data stored in the database. Authorized batch submission from third party registries through the RESTful Web service is only validated server-side. Third party registry software providers are informed to guarantee the integrity and validity of their data.” [1]

“The BCCR system includes validation components that prevent entering erroneous information by the users.” [4]

“The data is automatically validated during the data entry process.” [11]

“Data Quality

The software included automatic check features, such as logic branching and requiring a response, to ensure accuracy and completeness before being saved. During data collection, team supervisors accompanied enumerators and did spot checks to ensure that enumerators were filling in forms correctly. If any errors were found, they were corrected on the tablet if possible, or else recorded in an error log and reported to the Data Manager for resolution in the electronic database daily or at the end of each phase. During vaccination, data collected on vaccine recipients for the vaccine registries were linked directly with census data in the tablet records, allowing for accurate data linking at the point of vaccination. The registries were reviewed nightly or every two nights, and cleaned at the end of each vaccination phase.” [18]

“With REDCap, we restricted data format/type, set ranges for date and numeric fields, and allowed data validation. Data consistency problems such as incorrect data type, values out of range, and outliers for numerical fields can be reported using the data quality module.” [25]

Explanation

The system should have implemented checks to validate inserted data automatically. In this phase mainly range checks and missing data will be rejected. There should be hard and soft checks. While hard checks prevent the insertion of implausible values and don’t let the user continue until the implausible values are corrected, soft checks display a warning only and enable the user to continue, also if complained values will not be changed.

Item 6.10 The system has a query tool to perform automatic data queries.

Explanation

The system should have an integrated query tool that checks the data and performs automatic queries. These queries can be performed by the study groups and the answers will be inserted automatically in the database. With such a system it is possible to perform more sophisticated checks than during the input phase.

Item 6.11 The system has an interface for manual data check.

Example

> "CMR data quality assurance and quality control
>
> Web-based applications were developed to allow CMR staff to routinely perform data quality assurance and quality control (QA/QC) activities. For instance, the link on the main menu, CheckCMRSybase tables (right section in Figure 1), leads to applications that enable CMR staff to check invalid data and track referential integrity of links among CMR's relational data tables to make sure that no "orphan" records exist in the data tables." [26]

Explanation

Beside the automatic checks during data insertion and an automatic query tool, a patient registry software system should also provide an interface for manual data check. Not all errors can be detected by program and in some cases it is essential to check the data manually and perform individual queries.

Item 6.12 It is possible to perform manual data queries within the system.

Explanation

Since the data is checked manually it should also be possible to perform manual data queries within the system. These questions can be answered by the study centers and the correct data can be inserted directly in the system. Otherwise it may be too cumbersome to resolve manually detected errors if this must be done outside the system and results must be integrated later.

Item 6.13 Entries with unresolved queries are marked with flags at different levels (item, part, patient, etc.).

Example

> "While the monitor cannot change any data, he/she can flag reminders to the local supporting team when suspicious data is encountered such as large discrepancy in body mass index and waist circumference." [23]

Explanation

All entries with unresolved queries should be marked with flags at different levels (item, part, patient, etc.) to indicate the user that there are data to check. If the queries are answered these query flags should disappear.

Item 6.14 Implausible entries are marked with flags at different levels.

Example

> "The red, grey, yellow, and green icons signify incomplete, blank, unverified, and complete records, respectively. Clicking any of the colored buttons in the table automatically activates the associated data collection instrument." [25]

Explanation

All entries with implausible values should be marked at different levels with flags to indicate the user that these data must be checked and possibly corrected. This would improve data quality and completeness, so we recommend that a patient registry software system should provide this feature. As described by Pang et al. [25] in the above example the query flags should be connected with the associated data collection instrument, so the corrected values will be inserted directly in the database.

Item 6.15 Unplanned visits can be integrated flexibly.

Explanation

It should be possible to easily integrate unplanned visits in the system, so the data of additional visits can also be captured and are available in the system.

Item 6.16 The system should be designed following the standards of software ergonomics, defined in ISO 9241-110 [27].

Example

> "As pointed out in the introduction, CHES was designed to foster the integration of PROs in daily clinical routine. To develop an appropriate IT solution for this scenario, it is of utmost importance to constantly take the application's end users' feedback into account [26]. Usability prototyping and usability testing ensure that users are included at an early stage (standards for user oriented software are specified in ISO 13407)." [9]

Explanation

The standards of software ergonomics, as defined in ISO 9241-110 [27] should be adhered to when developing a new patient registry software system. As described by Holzner et al. [9], the end users should get involved in the development process early to evaluate the system, see also item 2.4.

7. Data Analysis (F)

This topic contains issues with which the registry software system can support data analysis.

Item 7.1 The system has a query builder to assist researchers to select interesting patient cohorts.

Example

> "The end user, typically a research investigator, accesses the registry through a web-based query interface. Following secure log in, the user encounters a graphical query builder interface in which registry-specific ontologies may be browsed; search terms may be dragged and dropped to construct queries to define subject cohorts of potential interest." [8]

Explanation

The system should provide a web-based interface with a query builder for researchers. As described by Natter et al. [8], in this query builder registry-specific ontologies may be browsed, search terms may be dragged and dropped to construct queries and select subject cohorts of potential interest for further analysis.

Item 7.2 The system is able to generate reports for selected cohorts.

Example

> “Selected cohorts are returned as patient sets, for which choices of pre-defined summary reports and visualizations may be generated in real time (figure 2).” [8]

Explanation

The system should be able to generate reports for selected cohorts. With such a component researchers can get easily an impression of the data in the registry, this is an important instrument to generate status reports during the project time and reports can be created to support decisions for further analysis of the data.

Item 7.3 Datasets can be generated and downloaded for analysis in different formats, complete or for selected cohorts.

Examples

> “Since the FRB! Project software is designed to be a research tool, data export and analysis features are very important. Individual Users can download their own data at any time as a text file in comma separated variable format. The software also offers statistical tools for simple analyses. Users can export their own data and analyze it as they see fit for more sophisticated analyses.” [6]

> “In this way, end users may iteratively refine their searches and are able, with appropriate authorizations, to download the resulting datasets for further analyses.” [8]

> “The database is kept and managed at the coordinating center, but data export and statistics are available at any time to every user within the SUN.” [10]

Explanation

It is important that datasets can be selected and downloaded for further analysis. This is necessary for analysis of the complete data and also for downloading specific cohorts. For example, in cooperative research projects it should be possible for each participating group to have access to their own data at any time.

Item 7.4 Results can be presented as colored graphs in real time.

Examples

> “The end user, typically a research investigator, accesses the registry through a web-based query interface. Following secure log in, the user encounters a graphical query builder interface in which registry-specific ontologies may be browsed; search terms may be dragged and dropped to construct queries to define subject cohorts of potential interest. Selected cohorts are returned as patient sets, for which choices of pre-defined summary reports and visualizations may be generated in real time (figure 2). In this way, end users may iteratively refine their searches and are able, with appropriate authorizations, to download the resulting datasets for further analyses.” [8]

> “Graphical presentation of results: results are presented as colored graphs in real time. The graphical output (see Figure 2) links PRO to the course of disease and treatment and in addition specific medical interventions can be easily incorporated and displayed. Results can be displayed optionally in a longitudinal or cross-sectional setup.” [9]

Explanation

It should also be possible to show results as colored graphs in real time within the system. This helps researchers get a quick overview and obtain an impression of the captured data in the system and make informed decisions for further analysis or continuation of the registry.

Item 7.5 The system gives interactive feedback, classifying the patient through based on implemented knowledge or scoring systems.

Examples

> "Flag System: Based on reference values from literature or previously collected data, the Flag System allows for the quick identification of patients with clinically relevant problems, using cut-off scores or score distributions." [9]

> "Clinicians were impressed by the automatic Revised Trauma Score calculator, which provided an instant estimate of survival probability once admission vital signs were entered. Early availability of prognostic scores for patients who are actively being resuscitated would represent a significant advance over North American injury scoring, in which data collection and entry by analysts are often required before these scores are available. Even more importantly, injury scoring can create the unprecedented possibility of comparing adjusted outcomes to national or international norms." [16]

> "Based on results estimated by the JADE Risk Engine, the e-portal displays the 5-year probability of major clinical events which can be adjusted by changing values of modifiable risk factors to promote discussions between patients and care providers (Figure 3). Data collected at each review visit are displayed to show the trends of control of modifiable risk factors including BP, HbA1c, LDLC and body weight. General recommendations can be triggered by predefined levels of risk factors to prompt care providers and patients to take appropriate actions. Printable reports showing risk predictions, trends of risk factor control and practice tips can be generated for care providers (in English) and patients (in 5 different Asian languages i.e. English, Thai, Korean, Malay and Chinese [both traditional and simplified Chinese]) for record purpose (Figure 4). Furthermore, the portal provides matrixes to help doctors monitor patients' levels of adherence to care processes (e.g. annual assessment, review visits, education sessions, laboratory tests) and self management as well as their status of attainment of treatment targets. These targets can be modified depending on the evolution of international healthcare standards." [23]

Explanation

The system should give interactive feedback according to the entered patient data to implemented knowledge bases and scoring systems. This is a great advantage of web-based information systems that patients can get immediate feedback when their data is entered into the system. They can take advantage of already available information at the beginning of their treatment. This is also a great advantage for physicians, they do not have to check each patient data by hand but they get the information from the system immediately after entering the patient data. Therefore the patient registry software system can help in knowledge-based decision making.

8. Security aspects (F)

This topic contains security aspects of the registry software system and important security aspects of the registry operation process.

The system must be secure according to the actual regulations.

Item 8.1 Only authorized users have access to the data.

Examples

> "The CNRDS system is only accessible to registered users at participating sites. To ensure that they have the authority to proceed with data entry, authorized users are issued their own unique electronic signature, i.e., a username/password combination." [1]

> "The BCCR web application that supports data collection is accessible only for authorized users, … . The authorized users must have their own unique electronic signature—a combination of a user name and a password." [4]

Explanation

Only authorized users may have access to the system. This must be guaranteed at least via an access control consisting of a username and password. In some cases it can be necessary to provide an additional secure component for example an identity card, but in most cases username and password should be sufficient.

Item 8.2 The system provides role-based user access.

Examples

"Each user has an appropriate level of access to data. The user roles and types of authority are described in Table 1." [4]

Table 1. BCCR user roles and their authority.

Role	Authority
Subject/patient	Can enter/update personal, demographic, lifestyle, symptoms, QOL, family and medical history data
Lab technician	Can enter/update biospecimen data only
Clinician	All of the above + Enter medical data, retrieve and edit existing cases of his/her patients
Coordinator	All of the above for the assigned clinicians
Center manager	All of the above + Retrieve and edit cases of the patients of the center/institution
System coordinator	All of the above + Retrieve and edit all cases, activate/suspend users, assign user authorities

"The spectrum of functionality offered to the logged-in user varies dependent on his/her role in the system; the predefined roles include study nurse, clinician, supervising clinician, data quality manager, and registry administrator." [11]

Explanation

Role-based user access is an important method to give several users different rights to the system and the data. A patient registry system should provide this feature because not all users need all rights. This is also an important feature to protect the data because some users require only the right to read the data and not to insert or change data. So unintended data changes can be prevented. The user roles can be created and allocated according to the specific needs of the project.

Item 8.3 The system utilizes secure web server communication through encrypted data transfer.

Examples

"The system utilizes Hypertext Transfer Protocol Secure (HTTPS) for web server communication." [1]

"The system utilizes secure web server communication and supports Secure Socket Layer (SSL) (an Internet encryption method that provides two-way encryption along the entire route that data travels to and from a user's computer) and Hypertext Transfer Protocol Secure (HTTPS) authentication (the communications standard used to securely transfer pages on the Web)." [4]

"All data transmissions between the user and the server are encrypted using 128-bit encryption (Secure Sockets Layer)." [6]

"Complete data transfer is encrypted using an industry standard (SSL/TLS: Secure Sockets Layer/Transport Layer Security)." [11]

Explanation

The complete data transfer must be encrypted according to actual transcription protocols and standards (SSL/TLS: Secure Sockets Layer/Transport Layer Security, with a secure transcription, for example Triple-DES or AES and the hash total SHA with high key length, 256 or 512), so if data are intercepted they are useless for the thief because they are encrypted and not readable.

Item 8.4 Sensitive Data can be stored encrypted in the database.

Examples

> "All subjects' identical information collected in the CNRDS is encrypted when entered into the database, which minimizes the risk of unauthorized access to this data." [1]

> "All Protected Health Information (PHI) is stored encrypted in the database and requires encryption/decryption functions with a pass phrase in order to insert or select data." [4]

Explanation

It should be possible to store sensitive data encrypted in the database. However, personal identification data should not be stored together with the medical data in the registry. Therefore we created item 6.1 pseudonymous patient identifier (PID). If it is necessary to store the identifying information, it should be stored in a separate database and it can be connected through the PID. Then it should be possible to store the identifying information encrypted in a separate database. However, beside the PID it might be possible that there is also medical information which requires encrypted storage in the database.

Item 8.5 All changes in the database are tracked and monitored through an audit trail.

Examples

> "The BCCR maintains an audit trail of all data entries to protect the authenticity, integrity and confidentiality of all data entries." [4]

> "*Audit Trails* implement a complete logging of any database transaction. Disregarding the data that is changed, all changes are logged in the same table build from the columns (i) User, (ii) Timestamp, (iii) Action, (iv) Revision, (v) Entity, (iv) Property, (v) DataType, (vi) OldValue and (vii) NewValue. The Action identifies whether new data has been created or existing has been modified (i.e., insert or update) and Revision is a counter that is incremented with the transaction. Hence, modifications in the database resulting from the same user action are labeled with the same revision number and can be easily joined. Entity and Property refer to the database table that has been modified and the according field, respectively." [5]

> "The electronic registry platform operating the Cochlear P-IROS contains an inherent audit trail to trace all amendments made with each form, the investigator making the change and when changes are made." [15]

> "Every interaction with the data is logged, creating an audit trail." [25]

Explanation

All data entries and changes should be maintained in an audit trail so that they can be tracked, and if necessary be reversed.

Item 8.6 If necessary, master-slave replication should be established.

Example

"We established MySQL master-slave replication for load-balance and, more importantly, for backup. Replication enables data from one MySQL database server (the master) to be replicated to one or more additional servers (the slaves). Because data is replicated to the slave(s), and the slave(s) can pause the replication process, it is possible to run backup services on the slave(s) without corrupting the corresponding master data [34]." [1]

Explanation

In some cases it might be useful to have a system which provides master-slave replication. If it is not sufficient to store backups regularly (item 8.7) it should be considered, master-slave replication may be a useful additional security component.

Item 8.7 Backups are stored separately regularly.

Example

"Further, a dumped database is packaged and transferred to a backup facility located in a different network zone every night." [1]

Explanation

Backups should be stored regularly, at least every night, so that if a system failure occurs it is possible to restore the system to the data status from the previous night and only the changes from the current day are lost.

Item 8.8 The server is behind a firewall.

Example

"The security system consists of both the infrastructure, policies, and protocols in place today, as well as novel functionality and approaches to support privacy and security protections including firewalls at strategic points that enforce access control policies between networks, and authentication and identity proofing procedures ensuring that only authorized users can access the application and data for which they are authorized." [26]

Explanation

The server must be located behind a firewall to minimize the risk for attacks.

Item 8.9 The server room is locked and temperature controlled.

Examples

"According to the HIMSS, a complete security solution that maximizes the benefits of networked data communications must contain the following elements: User Authentication, Access Control, Encryption, Physical Protection, and Management.[19]" [3]

"Every effort is made to ensure confidentiality by means of data encryption, password authentication of users, electronic firewalls and locked storage facilities, de-identification of PHI, audit trails, a disaster prevention and recovery plan, and security measures for back-up." [4]

Explanation

The server must be located in an access and temperature controlled room to minimize the risk for unwanted access and system failure through improper temperature.

9. **Privacy (F)**

This topic describes privacy aspects of the registry and of the software system additional to items 6.1 and 11.4.

Item 9.1 A data protection concept should be established before starting a registry project.

Example

> "Because the registry focuses also on the long-term outcome, patient identity is also obtained, which imposes high requirements on data privacy and protection. In this regard, the system's architecture and the data privacy and policy concept follow the generic framework of the German Technology, Methods and Infrastructure for Networked Medical Research (TMF) organization.6 The TMF and the state commissioner for data protection of Baden-Württemberg were both consulted regarding the concept. In addition, the data privacy and policy concept of CERTAIN was approved by the Ethics Committee of the University of Heidelberg. To reduce the amount of regulatory work for participating centers, the registry headquarters offers the service of providing the required documents to the respective local Ethics Committees." [11]

Explanation

Before starting a new registry project there should be a data protection concept established which takes into consideration all known use cases that may occur during the registry project.

Item 9.2 The registry software system should provide double pseudonymization for biological and genomic data.

Explanation

If biological data are integrated in the registry they should be double pseudonymized according to the concepts of the TMF e.V. for example [19] or using an algorithm such as specified in Prasser et al. [20], see also item 3.13.

10. General Features (F)

This topic contains general items which have no direct relation to the other topics.

Item 10.1 The costs of a patient registry software system must be taken into consideration, when choosing a software system: Is there a realistic calculation of the costs considering the costs of the procurement / programming, operation, life-cycle, archive, system replacement? Are the financial resources sufficient regarding the anticipated costs of sustainability of the registry guaranteed?

Examples

> "DADOS Prospective is a Web-based application developed by the research on research group (RoR) [37] to support data collection activities among researchers, research groups, and research networks [7]. It enables users to replicate any case report form into an eCRF, collect data in single/multisite studies, and extract data in an interoperable format. It is compliant with Chapter 11, Title 21, Code of Federal Regulations [4] and Health Privacy and Accountability Act (HIPAA) guidelines for EDC. It can be used to streamline and support individual/departmental/institutional databases, registries, and single/multisite clinical/nonclinical studies and clinical at a low cost [28]. … Cost is inarguably an important factor to be considered while choosing an EDC system. Although the nature of licensing and support required to maintain an EDC system are the main predictors of cost, the purpose of data capture and workflow at the site of implementation also have a major influence on the cost. Implementation at an institutional, departmental or individual level, number and type of users, single site or multi site data collection and workflow complexity are some examples of the latter. Commercial EDC systems like Oracle Clinical,

InForm and Rave are expensive in comparison to open source EDC systems like DADOS prospective, OpenClinica and TrialDB. While, the former have a higher presence in industry sponsored clinical trials, the latter are more common in academic settings." [28]

"The technological platform was notable for its low production cost." [29]

Explanation

As it is important to calculate and check the costs of a patient registry project, this is also true for the used system. Especially in universities research projects have to cope with limited financial resources and the costs for commercial software or outsourced software systems are not affordable. Besides this disadvantage, commercial software systems are often restrictive and in case of outsourced systems the data must be given elsewhere. So in university settings open source systems and own developments are predominant.

Item 10.2 The system has a multi-client capability. Several projects can simultaneously be executed in one installation of the system, but should be strictly separated.

Example

"The i2b2-based self-scaling registry platform (i2b2-SSR) developed for this purpose allows individual investigators and institutions to join a secure research data network by contributing a unique dataset and working with others to create larger, collaborative datasets that may be shared with the network as a whole or within specific subsets of sites and investigators." [8]

Explanation

If several projects can be handled in one installation of a registry software system this would reduce installation time and costs, and it would foster the cooperation within different registry projects. In the above example Natter et al. [8] describe the cooperation of different registries in one installation of a registry software system. Especially the different user-groups can benefit from such a solution, because they can share their data and increase the number of patients in each individual project.

Item 10.3 An update mechanism for the system is in place.

Examples

"Update Mechanism: In order to efficiently install updates for local CHES installations, an update mechanism is in place, providing an one-click solution for installing, new features and available bug fixes." [9]

"An automatic update function is also highly recommended for future software upgrades." [13]

Explanation

There should be an update function for the software which allows easy installation of future software updates. So the system can be updated when a new version is available and new features or security aspects can easily be installed.

Item 10.4 Source documentation of CRFs in pdf format is possible.

Example

"The system must be integrated in the existing physician's workflow. Therefore, this electronic registration is almost identical as paper-based registration. Instead of the paper forms, data are entered in a PDF, which can be processed by a computer. If the physician still wants to keep a paper-based print-out, an identical paper will be produced, while the system stores the data entries for long-term analysis." [12]

Explanation

Sometimes the study team requires that the CRFs with the source values can also be extracted as PDF for offline use. If this feature is needed it should be considered when the software for the system is chosen.

11. Organizational (F)

This topic comprises software-related organizational items.

Item 11.1 The system is compliant with all known relevant regulations.

Example

> "In this regard, the system's architecture and the data privacy and policy concept follow the generic framework of the German Technology, Methods and Infrastructure for Networked Medical Research (TMF) organization.[6] The TMF and the state commissioner for data protection of Baden-Württemberg were both consulted regarding the concept. In addition, the data privacy and policy concept of CERTAIN was approved by the Ethics Committee of the University of Heidelberg. To reduce the amount of regulatory work for participating centers, the registry headquarters offers the service of providing the required documents to the respective local Ethics Committees." [11]

Explanation

The patient registry and also the used software system must be compliant with all known relevant regulations which are applicable in the specific governing area. The regulations may vary from area to area, so it has to be checked which regulations are applicable and it must be ensured, that the patient registry and the used software system are compliant with all these regulations.

Item 11.2 The registry is compliant with Chapter 11, Title 21, Code of Federal Regulations and HIPPA.

Examples

> "IRB and subject recruitment
>
> The BCCR participating centers are required to obtain approval from its Institutional Review Board (IRB). The BCCR provides standard protocol templates and privacy assurances in procedures of informed consent that have been formulated to detail the use of web-based tools. A template of common protocol statements includes: (i) methods and procedures applied to human subjects; (ii) data storage and confidentiality; (iii) potential risk assessment for human subjects; (iv) risk classification; (v) protection against the potential risks for human subjects; (vi) potential benefit assessment for human subjects; (vii) potential benefits to society; and (viii) alternatives to participation. A template for a common informed consent form includes the following HIPAA-mandated information: (i) a specific description of the information to be used or disclosed; (ii) the person or entity to whom disclosure will be made; (iii) the purpose of the use or disclosure; (iv) an expiration date or event for use of the information; (v) an explanation of how authorization may be revoked; and (vi) any restrictions placed on the subject's access to the information with access granted upon completion of the research. All participating investigators are able to use these standardized statements to assist them with their IRB applications.
>
> All BCCR participating researchers and clinicians are required to complete the computer-based training course on the Protection of Human Research Subjects. All information gathered in the BCCR should be compliant with IRB approvals at participating sites that are monitored by each center's IRB. The BCCR coordinator opens new accounts and enables data entry into the BCCR only after receiving the documented proof of IRB protocol approval. In order to enter data into the BCCR, a copy of the consent form for each subject must be submitted to the BCCR coordinator. Under the informed consent process,

study participants have been asked to voluntarily participate in the BCCR. The potential participants are asked about their willingness to share the information they provided in the BCCR with research collaborators. The information the participants provide is collected for research purposes only. The subjects are informed in the consent that their PHI will be encrypted and that the web-based registry is accessible to authorized users only. Identifiers will never be released in order to protect participant confidentiality. Every effort is made to ensure confidentiality by means of data encryption, password authentication of users, electronic firewalls and locked storage facilities, de-identification of PHI, audit trails, a disaster prevention and recovery plan, and security measures for back-up.

Participants have also been informed that they may revoke the authorization to use and share their PHI at any time by contacting the principal investigator in writing. If they revoke the authorization, they may no longer participate in the research studies and the use or sharing of future PHI will be stopped, but the PHI which has already been collected may still be used." [4]

"DADOS Prospective, OpenClinica1 and Redcap are examples of open source EDC systems. DADOS Prospective is a Web-based application developed by the research on research group (RoR) [37] to support data collection activities among researchers, research groups, and research networks [7]. It enables users to replicate any case report form into an eCRF, collect data in single/multisite studies, and extract data in an interoperable format. It is compliant with Chapter 11, Title 21, Code of Federal Regulations [4] and Health Privacy and Accountability Act (HIPAA) guidelines for EDC. It can be used to streamline and support individual/departmental/ institutional databases, registries, and single/multisite clinical/nonclinical studies and clinical at a low cost [28]." [28]

Explanation

The patient registry software system and the complete data workflow and data storage should be compliant with Chapter 11, Title 21, Code of Federal Regulations and HIPPA to ensure high quality research. If necessary, informed consent must be obtained from the patients.

Item 11.3 There are clearly defined rules which describe the rights on the data for each participating institution.

Examples

"Organizational model

The BCCR utilizes the confederation model assuring that each institution voluntarily participates in the registry, retains all rights to its own data, and has equal representation in the registry's steering committee. A confederation encourages any interested center regardless of its size or location to participate in database development and utilization. The data collected at any location can be used by other participants only after obtaining required permissions and by providing corresponding references and acknowledgements." [4]

"Since the FRB! Project software is designed to be a research tool, data export and analysis features are very important. Individual Users can download their own data at any time as a text file in comma separated variable format. The software also offers statistical tools for simple analyses. Users can export their own data and analyze it as they see fit for more sophisticated analyses." [6]

"The steering committee created bylaws and rules of procedure for the registry, which clearly describe the ownership of the stored data and the rights for analyses and publications. The data provided by each participating center can be exported directly from the CERTAIN web application by this particular center without involvement of the registry headquarters or the steering committee. Similar rights are granted on a national level for the participating countries. Country-specific analyses are facilitated by a national coordinator, who primarily interacts with the registry's headquarters. Only analyses of the multinational dataset in the CERTAIN Registry require approval by the steering committee." [11]

Explanation

The registry should have a steering committee as described by Plotnicki et al. [11], which defines among other things the rights on the data in the registry. It should be regulated that each participating institution receives a copy of their inserted data at any time. It would be preferable that they can download a copy of their own data at any time without contacting the registry headquarters.

Item 11.4 The system is compliant with all known appropriate data protection guidelines of the registry project.

Example

> "In this regard, the system's architecture and the data privacy and policy concept follow the generic framework of the German Technology, Methods and Infrastructure for Networked Medical Research (TMF) organization.[6] The TMF and the state commissioner for data protection of Baden-Württemberg were both consulted regarding the concept. In addition, the data privacy and policy concept of CERTAIN was approved by the Ethics Committee of the University of Heidelberg. To reduce the amount of regulatory work for participating centers, the registry headquarters offers the service of providing the required documents to the respective local Ethics Committees." [11]

Explanation

A data protection concept should be formulated and the patient registry software system, the complete data workflow, and data storage should be compliant with all known according data protection guidelines.

12. Training (D)

This topic contains items which are important for the user training.

Item 12.1 There should be manuals for the registry end-users and for the operators.

Examples

> "The comprehensive training materials, manuals defining vocabulary used in the BCCR and user manuals with a defined set of procedures and lines of responsibilities for each level of participants were distributed to the centers." [4]

> "A quick tutorial on how to use CIRIS was created by the SIT and placed on the laptops and in a project binder next to these computers." [12]

Explanation

A user manual how to use the system should be available for the operators, and there should be also one with project specific content for all end-users involved in the registry project.

Item 12.2 At the beginning, and if necessary during the project time, a training is provided for the users.

Examples

> "To guarantee the consistency and reliability of data collection across the participating centers, the BCCR coordinator continuously provides educational training sessions and audits submitted data, whereas individual center managers review the data submitted from their respective centers." [4]

> "Personnel Training for Data Collection

We performed a semi-structured training with the clinical research coordinators. Our goal was to provide a general overview of the registry database, while concurrently identifying specific factors which could compromise the integrity of the data collection. To ensure a standardized and consistent data collection we developed a standard operating procedure (SOP) specifically related to the primary data collectors tasks. This SOP provides a description of all data elements collected as well as the sources used to obtain the data. After the training process, the data entry activities of clinical research coordinators were closely monitored for three months by the principal investigators (RC and KRS) to assess whether data collection was conducted according to the study protocol. We used the REDCap report tool for monitoring and querying patient records. Corrective actions were taken to address problems related to data inconsistency and missing information, involving retraining and immediate feedback on issues such as missing, out-of-range values and logical inconsistencies." [17]

Explanation

At the beginning, and if necessary during the project time, a user training should be provided to instruct the end-users how to use the system. The training should also include project specific content to minimize data errors caused by wrong insertion. Also for the operators, appropriate training sessions should be provided.

Item 12.3 Regularly user feedback is collected for further improvements of the system.

Examples

"The BCCR developers regularly collect feedback from the end users and evaluate the system's interface for further improvements." [4]

"The incident reporting screen is the cornerstone of the safety improvement initiative and will evolve over time on the basis of user feedback." [30]

"P-PROMPT Implementation, Training, and Impact

The primary health care team's appraisals of P-PROMPT were examined via questionnaire with respect to the following domains: Learning, Training, Using, Usefulness, Daily Practice, Practice Planning, CDMS, Support from the Service Provider, and Satisfaction. Each domain was evaluated using several questions. All questions were phrased using a 5-point Likert scale in a positive direction, where *completely agree* was a positive response and *completely disagree* was a negative response (Appendix 1). All physicians enrolled in the study were asked to complete periodic questionnaires, at 2 months, 6 months, and 12 months." [31]

Explanation

We recommend collecting regularly user feedback for further improvements of the patient registry software system. Like in O'Reilly et al. [31], the collected data can be analyzed and it can be shown if the used system had an influence on the disease management. These data can also be used to improve further projects for example like O'Reilly et al. concluded for user trainings how to use a system at the beginning of a new project, [31].

Item 12.4 The system provides an online help for data entry.

Example

"An on-line guide has been designed to help the users in better understanding the meaning of the information to be entered. Its contents have been structured at three different levels, namely context, input form, and single item.

Context-related material explains the rationale for collecting a set of data which can be entered through one or more forms. For example, SUN registry data belong to the "stroke" context, that can be further

refined into narrower contexts such as *emergency, SU admission, discharge and follow-up*. Form-specific help instructs the user about the intents and the features of each input form, relating those to the specific context they belong to. Finally, item-related help addresses any possible ambiguity about the meaning of a specific form item. …

We often added explanatory labels for date and time data, which is very important as some data may be associated to different timestamps. …

In addition to plain explanations used by the on-line help, we also explicitly represented any relationship, based on medical knowledge, useful for detecting treacherous data entry errors …

These relationships have been organized into a semantic network, and are exploited to generate warnings whenever the actual data violate them. …" [10]

Explanation

An online help is an innovative instrument to support end-users in the data entry process. It is immediately available when questions during the data entry process appear and it is more often used than a written manual because it is available on-site. When contexts are organized in a semantic network to provide online help, as described by Lanzola et al. [10], this can help to prevent serious errors which otherwise will not be detected. For example, if a medication is applied which is not related to the disease this can be detected by an online help when contexts are organized in a semantic network. Such errors would otherwise not be detected by simple range checks. We recommend implementing such an online help in patient registry software systems.

References

[1] Xie F, Zhang D, Wu J, Zhang Y, Yang Q, Sun X, Cheng J, Chen X. Design and implementation of the first nationwide, web-based Chinese Renal Data System (CNRDS). BMC Med Inform Decis Mak. 2012;12:11. doi: 10.1186/1472-6947-12-11.

[2] Messiaen C, Le Mignot L, Rath A, Richard JB, Dufour E, Ben Said M, Jais JP, Verloes A, Le Merrer M, Bodemer C, Baujat G, Gerard-Blanluet M, Bourdon-Lanoy E, Salomon R, Ayme S, Landais P. CEMARA: a Web dynamic application within a N-tier architecture for rare diseases. Stud Health Technol Inform. 2008;136:51-6.

[3] Sherman S, Shats O, Ketcham MA, Anderson MA, Whitcomb DC, Lynch HT, Ghiorzo P, Rubinstein WS, Sasson AR, Grizzle WE, Haynatzki G, Feng J, Sherman A, Kinarsky L, Brand RE. PCCR: Pancreatic Cancer Collaborative Registry. Cancer Inform. 2011;10:83-91.

[4] Sherman S, Shats O, Fleissner E, Bascom G, Yiee K, Copur M, Crow K, Rooney J, Mateen Z, Ketcham MA, Feng J, Sherman A, Gleason M, Kinarsky L, Silva-Lopez E, Edney J, Reed E, Berger A, Cowan K. Multicenter breast cancer collaborative registry. Cancer Inform. 2011;10:217-26.

[5] Deserno TM, Haak D, Brandenburg V, Deserno V, Classen C, Specht P. Integrated Image Data and Medical Record Management for Rare Disease Registries. A General Framework and its Instantiation to the German Calciphylaxis Registry. J Digit Imaging. 2014. doi: 10.1007/s10278-014-9698-8.

[6] Gillies MC, Walton R, Liong J, Arnold JJ, McAllister I, Morlet N, Hunyor A, Guymer R, Keeffe J, Essex R, Herrera-Bond A, Glastonbury B, Simpson JM, Barthelmes D. Efficient capture of high-quality data on

outcomes of treatment for macular diseases: the fight retinal blindness! Project. Retina. 2014;34(1):188-95.

[7] Singh SK, Malik A, Firoz A, Jha V. CDKD: a clinical database of kidney diseases. BMC Nephrol. 2012;13:23 doi: 10.1186/1471-2369-13-23.

[8] Natter MD, Quan J, Ortiz DM, Bousvaros A, Ilowite NT, Inman CJ, Marsolo K, McMurry AJ, Sandborg CI, Schanberg LE, Wallace CA, Warren RW, Weber GM, Mandl KD. An i2b2-based, generalizable, open source, self-scaling chronic disease registry. J Am Med Inform Assoc. 2013;20(1):172-9. doi: 10.1136/amiajnl-2012-001042.

[9] Holzner B, Giesinger JM, Pinggera J, Zugal S, Schöpf F, Oberguggenberger AS, Gamper EM, Zabernigg A, Weber B, Rumpold G. The Computer-based Health Evaluation Software (CHES): a software for electronic patient-reported outcome monitoring. BMC Med Inform Decis Mak. 2012;12:126. doi: 10.1186/1472-6947-12-126.

[10] Lanzola G, Parimbelli E, Micieli G, Cavallini A, Quaglini S. Data quality and completeness in a web stroke registry as the basis for data and process mining. J Healthc Eng. 2014;5(2):163-84.

[11] Plotnicki L, Kohl CD, Höcker B, Krupka K, Rahmel A, Pape L, Hoyer P, Marks SD, Webb NJ, Söylemezoglu O, Topaloglu R, Szabo AJ, Seeman T, Marlies Cornelissen EA, Knops N, Grenda R, Tönshoff B. The CERTAIN Registry: a novel, web-based registry and research platform for pediatric renal transplantation in Europe. Transplant Proc. 2013;45:1414-7.

[12] Steurbaut K, De Backere F, Keymeulen A, De Leenheer M, Smets K, De Turck F. NEOREG: design and implementation of an online Neonatal Registration System to access, follow and analyse the data of newborns with congenital cytomegalovirus infection. Inform Health Soc Care. 2013;38(3):223-35.

[13] Lycett K, Wittert G, Gunn J, Hutton C, Clifford SA, Wake M. The challenges of real-world implementation of web-based shared care software: the HopSCOTCH Shared-Care Obesity Trial in Children. BMC Med Inform Decis Mak. 2014;14:61.

[14] Wake M, Lycett K, Clifford SA, Sabin MA, Gunn J, Gibbons K, Hutton C, McCallum Z, Arnup SJ, Wittert G. Shared care obesity management in 3-10 year old children: 12 month outcomes of HopSCOTCH randomised trial. BMJ. 2013 Jun 10;346:f3092.

[15] Sanderson G, Ariyaratne TV, Wyss J, Looi V. A global patient outcomes registry: Cochlear paediatric implanted recipient observational study (Cochlear(™) P-IROS). BMC Ear Nose Throat Disord. 2014;14:10. doi: 10.1186/1472-6815-14-10.

[16] Zargaran E, Schuurman N, Nicol AJ, Matzopoulos R, Cinnamon J, Taulu T, Ricker B, Garbutt Brown DR, Navsaria P, Hameed SM. The electronic Trauma Health Record: design and usability of a novel tablet-based tool for trauma care and injury surveillance in low resource settings. J Am Coll Surg. 2014;218(1):41-50.

[17] da Silva KR, Costa R, Crevelari ES, Lacerda MS, de Moraes Albertini CM, Filho MM, Santana JE, Vissoci JR, Pietrobon R, Barros JV. Glocal clinical registries: pacemaker registry design and implementation for global and local integration--methodology and case study. PLoS One. 2013;8(7):e71090. doi: 10.1371/journal.pone.0071090.

[18] Teng JE, Thomson DR, Lascher JS, Raymond M, Ivers LC. Using Mobile Health (mHealth) and Geospatial Mapping Technology in a Mass Campaign for Reactive Oral Cholera Vaccination in Rural Haiti. PLoS Negl Trop Dis. 2014;8(7):e3050. doi: 10.1371/journal.pntd.0003050.

[19] TMF e.V. 2015. http://www.tmf-ev.de/ (accessed: 15 Apr 2016).

[20] Prasser F, Kohlmayer F, Lautenschläger R, Kuhn KA. ARX - A Comprehensive Tool for Anonymizing Biomedical Data. AMIA Annual Symposium Proceedings. 2014;2014:984-993.

[21] National Cancer Institute. The caBIG program has been retired. https://cabig.nci.nih.gov/. (accessed 12 Feb 2015).

[22] National Cancer Institute. National Cancer Informatics Program. 2015. http://cbiit.nci.nih.gov/ncip. (accessed 15 Apr 2016).

[23] Ko GT, So WY, Tong PC, Le Coguiec F, Kerr D, Lyubomirsky G, Tamesis B, Wolthers T, Nan J, Chan J. From design to implementation--the Joint Asia Diabetes Evaluation (JADE) program: a descriptive report of an electronic web-based diabetes management program. BMC Med Inform Decis Mak. 2010;10:26. doi: 10.1186/1472-6947-10-26.

[24] Zaman B, Khandekar R, Al Shahwan S, Song J, Al Jadaan I, Al Jiasim L, Owaydha O, Asghar N, Hijazi A, Edward DP. Development of a web-based glaucoma registry at King Khaled Eye Specialist Hospital, Saudi Arabia: a cost-effective methodology. Middle East Afr J Ophthalmol. 2014;21(2):182-5.

[25] Pang X, Kozlowski N, Wu S, Jiang M, Huang Y, Mao P, Liu X, He W, Huang C, Li Y, Zhang H. Construction and management of ARDS/sepsis registry with REDCap. J Thorac Dis. 2014;6(9):1293-9. doi: 10.3978/j.issn.2072-1439.2014.09.07.

[26] Wang Y, Tao Z, Cross PK, Le LH, Steen PM, Babcock GD, Druschel CM, Hwang SA. Development of a web-based integrated birth defects surveillance system in New York State. J Public Health Manag Pract. 2008;14(6):E1-E10. doi: 10.1097/01.PHH.0000338377.83736.9c.

[27] Ergonomic requirements for office work with visual display terminals (VDTs) - Part 11: Guidance on usability (ISO 9241–11:1998), 1998. http://www.it.uu.se/edu/course/homepage/acsd/vt09/ISO9241part11.pdf. (accessed 15 Apr 2016).

[28] Shah J, Rajgor D, Pradhan S, McCready M, Zaveri A, Pietrobon R. Electronic data capture for registries and clinical trials in orthopaedic surgery: open source versus commercial systems. Clin Orthop Relat Res. 2010;468(10):2664-71.

[29] Hira AY, Lopes TT, de Mello AN, Filho VO, Zuffo MK, de Deus Lopes R. Establishment of the Brazilian telehealth network for paediatric oncology. J Telemed Telecare. 2005;11 Suppl 2:51-2.

[30] France DJ, Miles P, Cartwright J, Patel N, Ford C, Edens C, Whitlock JA. A chemotherapy incident reporting and improvement system. Jt Comm J Qual Saf. 2003;29(4):171-80. Erratum in: Jt Comm J Qual Saf. 2003;29(5):209.

[31] O'Reilly DJ, Bowen JM, Sebaldt RJ, Petrie A, Hopkins RB, Assasi N, MacDougald C, Nunes E, Goeree R. Evaluation of a chronic disease management system for the treatment and management of diabetes in primary health care practices in Ontario: an observational study. Ont Health Technol Assess Ser. 2014;14(3):1-37.
